# Exploring the Possibilities of Using Recovered Collagen for Contaminants Removal—A Sustainable Approach for Wastewater Treatment

**DOI:** 10.3390/polym16202923

**Published:** 2024-10-18

**Authors:** Annette Madelene Dancila, Magdalena Bosomoiu

**Affiliations:** Department of Analytical Chemistry and Environmental Engineering, Faculty of Chemical Engineering and Biotechnologies, National University of Science and Technology Politehnica Bucharest, 7 Polizu Street, 011061 Bucharest, Romania; madelene.dancila@upb.ro

**Keywords:** circular economy, wastewater treatment, recovered collagen

## Abstract

Collagen is a non-toxic polymer that is generated as a residual product by several industries (e.g., leather manufacturing, meat and fish processing). It has been reported to be resistant to bacteria and have excellent retention capacity. However, the recovered collagen does not meet the requirements to be used for pharmaceutical and medical purposes. Due to the scarcity of water resources now affecting all continents, water pollution is a major concern. Another major field that could integrate the collagen generated as a by-product is wastewater treatment. Applications of collagen-based materials in wastewater treatment have been discussed in detail, and comparisons with already frequently used materials have been made. Over the last years, collagen-based materials have been tested for removal of both organic (e.g., pharmaceutical substances, dyes) and inorganic compounds (e.g., heavy metals, noble metals, uranium). They have also been tested for the manufacture of oil-water separation materials; therefore, they could be used for the separation of emulsified oily wastewater. Because they have been analysed for a wide range of substances, collagen-based materials could be good candidates for removing contaminants from wastewater streams that have seasonal variations in composition and concentration. The use of recovered collagen in wastewater treatment makes the method eco-friendly and cost efficient. This paper also discusses some of the challenges related to wastewater treatment: material stability, reuse and disposal. The results showed that collagen-based materials are renewable and reusable without significant loss of initial properties. In the sorption processes, the incorporation of experiments with real wastewater has demonstrated that there is a significant competition among the substances present in the sample.

## 1. Introduction

Following the increasing climatic changes, ensuring the availability of necessary water has become a major global emergency. Water is an important resource that is not available in all countries; restricted access to clean water limits socio-economic development and accentuates poverty. All the activities that take place, in industry, energy production, agriculture, leisure and everyday life, lead to water consumption and implicitly to the generation of wastewater (municipal, industrial, agricultural, a mixture of those listed previously). Depending on its source, the wastewater can be contaminated not only with secondary by-products but also with leached compounds (e.g., oil refinery wastewater also contains lead, nickel, chromium and iron that are leached from the pipes, equipment or catalysts used in the refinery [1]). High adsorption capacity towards heavy metals, dyes, vitamin C and p-nitrophenol was reported for a collagen-based hydrogel, suggesting its applicability for depolluting wastewater that contains a vast range of contaminants [2]. In the textile industry, which is a major contributor to water contamination [3], dye removal is usually accompanied by the need for simultaneous removal of heavy metals [4]. Collagen is often included in the formulation of materials that are both antimicrobials and adsorbents. In this way, the concentration of contaminants is reduced, at the same time decreasing the number of microorganisms present in the wastewater [5]. Coal gasification, which is still largely used in countries from Asia and in the United States of America, is another process that generates large amounts of wastewater; this water is charged with difficult-to-remove organic compounds that have mutagenic and carcinogenic characteristics. [6]. Wastewater is not charged only with chemical compounds but also with microorganisms that can be pathogenic [7].

Latest statistics and reports released by the United Nations Environment Programme warn about the significant quantities of wastewater that are discharged in water bodies without being treated: nearly two million tons of wastewater, consisting of sewage and agricultural and industrial wastewater, are released yearly [8,9]. From that, about 50% is directly discharged into the environment without preliminary treatment [10]. The United Nations have adopted a set of measures applied with the purpose of reducing the amount of untreated wastewater on one side and increasing the reuse of treated wastewater (e.g., in irrigation, as a cooling agent) [11].

Large amounts of waste rich in collagen are disposed by the leather manufacturing and meat and fish processing industries; in the context of a circular economy, collagen can be efficiently extracted to be reused [12,13]. For example, in the leather processing industry, raw hide has been reported to contain about 28% collagen; the mass percentage of collagen in trimmings is about 18%. Collagen mass balance in a leather production facility indicated that almost half of the collagen in the leather ends up disposed as waste (of 304 kg of collagen, 155 kg is found in the leather processing solid waste) [14]. Yearly, about 9 MT raw skin is processed, which in turn generates, according to the above given percentages, about 1.25 MT of collagen waste [15].

Attempts to use collagen in wastewater treatment have been made since 1995 when collagen was tested as a coagulant for sludge dewatering; the authors of the study proposed the use of collagen fibres together with alum ((Al_2_(SO_4_)_3_·18H_2_O)) for liquid-solid separation processes and sludge dewatering [16]. A later study also confirmed the ability to use collagen in composite flocculants with increased flocculation ability, compared to aluminium sulphate [17]. Reusing the collagen in wastewater treatment not only will provide a sustainable solution for waste minimisation but will also contribute to obtaining a valuable resource: water. The advantages that collagen brings as a potential component of the materials used in a wastewater plant (e.g., adsorbents, membranes, filtering material, catalysts) are as follows:

- Strength and flexibility provided by its structure [18];
- Ability to interact with a large number of chemical substances due to increased number of functional groups (amino, carboxyl, hydroxyl);
- No toxicity at low concentrations [19] (in the case of leaching in the treated water, will pose no health risk);
- Resistance to bacteria [20];
- Biodegradability [21,22].

Collagen’s ability to interact with different substances can be used in two directions: collagen can be easily incorporated in different materials (adsorbents, membranes, catalysts), and collagen will create bonds with contaminants present in the wastewater.

## 2. Collagen-Based Materials for Wastewater Treatment

**Figure 1:**
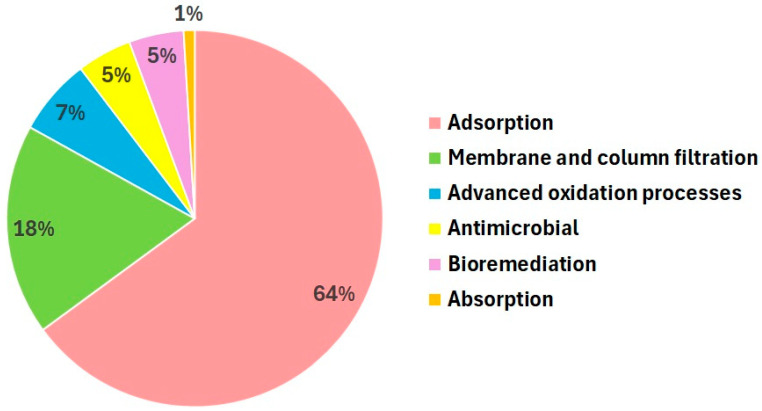
Studies on collagen’s use in the wastewater treatment.

In wastewater treatment, collagen-based materials have been tested in the processes of adsorption, membrane separation, column filtration, column absorption and advanced oxidation processes (photocatalysis and Fenton-like oxidation) and as components of antimicrobial materials. However, most of the studies are focusing on the use of collagen in adsorbents and separation materials (Figure 1). All these technologies are used in the secondary or tertiary stages of wastewater treatment [23]. The tertiary stage is considered the stage that brings the wastewater parameters to the levels set by the wastewater reuse directives. To achieve an optimal management of water resources and to reduce the water deficit, more and more treatment plants should be adapted by including the tertiary treatment stage.

### 2.1. Adsorption Technology

Adsorption is used in wastewater treatment plants due to the ease of operation and possibility of using a wide range of materials that can be adapted according to the type of contaminants (e.g., activate carbon synthesised from different waste materials, natural materials: zeolites, clay minerals). The cost of the material remains, however, a major disadvantage. In addition, the load factor of the adsorbent is an important parameter; a low load factor results in a larger volume of adsorbent in the column [24].

To improve the efficiency of the adsorption process, different materials containing collagen have been tested for wastewater treatment. The adsorbent materials need to fulfil at least the following requirements: high adsorption capacity, non-toxic, reusable for extended period and low cost. The adsorption is currently used in the tertiary stage of wastewater treatment along with photocatalysis, catalytic oxidation, electro-chemical oxidation, ozonation or membrane separation [25].

The studies published so far have evidenced that collagen not only serves as a support for the active compounds of the adsorbent but also creates bonds with the adsorbate, enhancing the adsorption capacity. The multitude of functional groups of the collagen matrix, such as -NH_2_, -COOH, -C(O)NH_2_ or -OH, determines its ability to interact with different contaminants (e.g., heavy metal ions, radioactive ions, etc.) and remove them from wastewater [26,27,28]. It has been demonstrated that some heavy metal ions, in the presence of polypeptides, form a three-helix bundle structure [29,30]. This gives stability to the newly formed structure, explaining the good adsorption capacity of collagen-based materials towards heavy metal ions. The high adsorption of Cr(III) was attributed to the additional carboxyl groups created by modifying the collagen structure with oxazolidine [31]. Zhao et al. (2024) found that the phenolic hydroxyl groups present in the tannin extract in the composite collagen/tannin were responsible for the high affinity towards Cu(II) ions [32].

To improve the properties of the final adsorbent, some of the collagen-based adsorbents investigated so far for wastewater depollution are crosslinked with synthetic polymers [33,34,35,36,37,38,39]. The use of nanocomposites is preferred because they provide a higher internal surface area available for contaminants adsorption [26,39,40,41,42].

A significant number of studies have been dedicated to heavy metals, noble metals and metalloids adsorption on collagen-based adsorbents [31,32,33,34,43,44,45,46,47,48,49,50,51,52,53,54,55,56,57,58,59].

**Figure 2:**
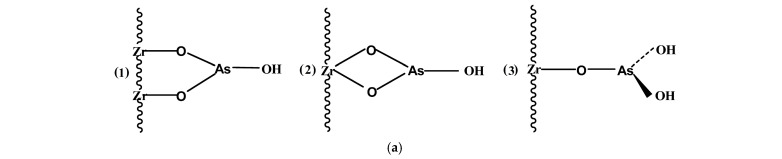

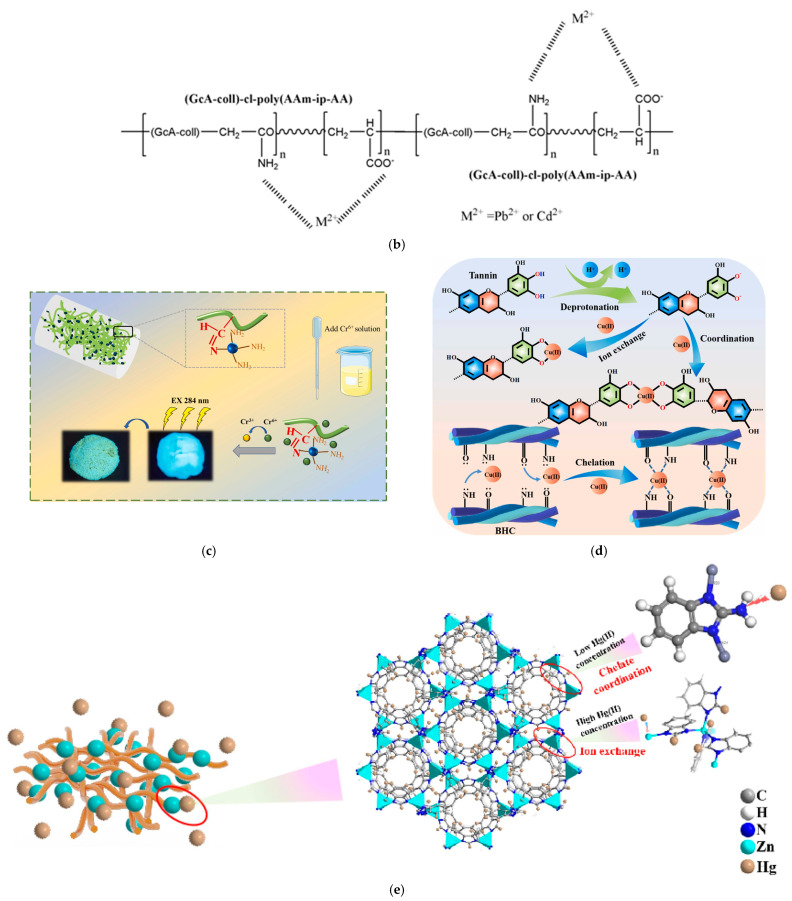
Mechanisms of heavy metals and metalloids ions adsorption on collagen-based materials; (**a**);As(III) removal by Zr(IV)—loaded collagen fibre [60]; (**b**) Removal of Pb(II) and Cd(II) over gum copal-collagen hybrid adsorbent [45]; (**c**) Cr (VI) adsorption by collagen-based porous fluorescent aerogel [56]; (**d**) Cu(II) retention on bovine hide collagen/tannin extract composite (BHC/TE) [32]; (**e**) Hg(II) retention on amino functionalized collagen fibre combined with metal–organic frameworks (MOFs) [57].

The enhanced removal of As(III) on Zr(IV) loaded on collagen fibres (CF) was explained by the ability of As(III) to form different configurations of complexes with zirconium-supported collagen fibres (Figure 2a): (1) bidentate, binuclear As(III) connects through oxygen with two zirconium ions; (2) bidentate, mononuclear As(III) forms two bonds with one zirconium ion via the oxygen ions; (3) monodentate, mononuclear As(III) creates one bond with one zirconium ion. This mechanism was similar to As(III) adsorption on alumina [60].

It was found that adjacent hydroxyl groups play a significant role in the retention mechanism. Removal of silver ions by persimmon tannins/CF also proceeds via a complexation between silver ions and adjacent hydroxyl groups of the adsorbent. Furthermore, the hydroxyl groups of the persimmon tannins oxidize to carbonyl groups, while the silver ions are reduced to elemental silver that is uniformly dispersed at the surface of the adsorbent [59].

The functional groups—NH_2_ (Lewis base) and -COO^−^ enhance Pb(II) and Cd(II) removal by coordination, respectively electrostatic attractions (Figure 2b); to increase the number of functional groups, gum copal CF was cross-linked with polyacrylic acid [45]. Similarly, Cr(VI) is adsorbed from wastewater on collagen-based porous aerogel; the ions of Cr diffuse in the porous structure of the adsorbent to coordinate with amino, hydroxyl and carboxyl groups (Figure 2c); since the synthesised aerogel had fluorescent properties, it can also serve as an indicator of Cr(VI) concentration [56].

To increase the number of adjacent hydroxyl groups, Zhao et al. (2024) synthesised a composite bovine hide collagen/tannin extract; the first step in Cu(II) removal consists of deprotonation of two adjacent hydroxyl groups of the tannin, followed by the creation of bonds due to electrostatic attractions (Figure 2d); the other functional groups coordinate with Cu(II) to form stable structures [32].

For Hg(II) adsorption on CF/ZIF-7-NH_2_ (ZIF—zinc imidazolate framework), it has been discovered that at low adsorbate concentration in wastewater, the removal mechanism is governed by electrostatic interactions and coordination, while at high Hg(II) concentration, the contribution of coordination is insignificant, and the Hg(II) adsorption takes place mainly by electrostatic interactions (Figure 2e) [57].

**Figure 3:**
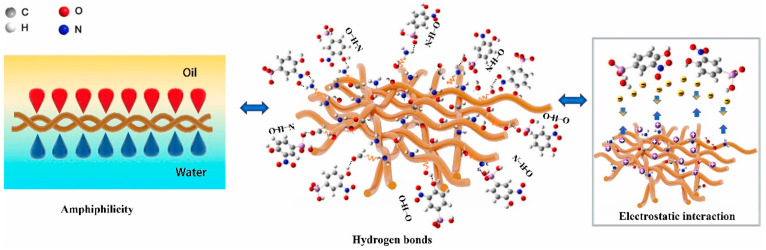
Mechanisms of organic pollutants adsorption on collagen-based materials—adsorption of p-arsanilic acid on amphiphilic amino modified collagen fibre [35].

Collagen fibre was modified with polyethyleneimine (PEI) and used for p-arsanilic acid adsorption (Figure 3); the obtained adsorbent has an increased capacity for contaminant removal via the combined effect of collagen amphiphilicity, formation of hydrogen bonds and electrostatic interactions [35].

**Figure 4:**
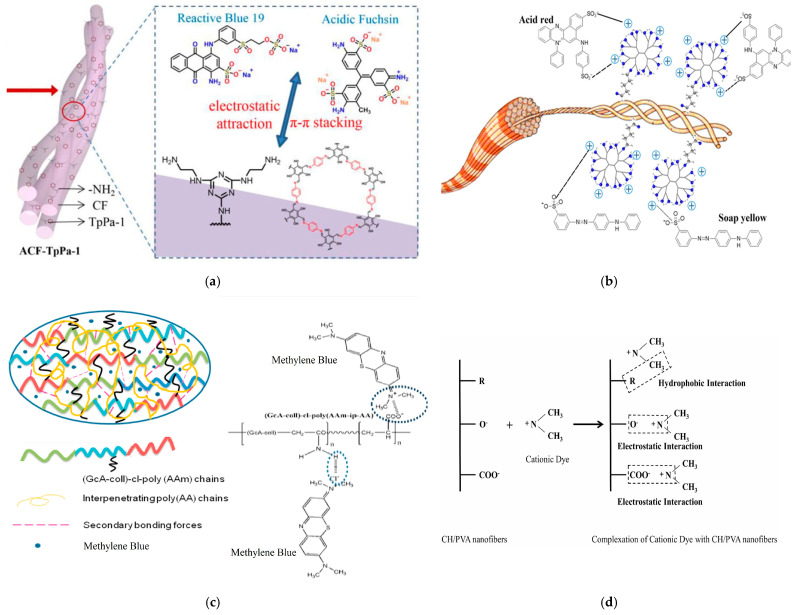

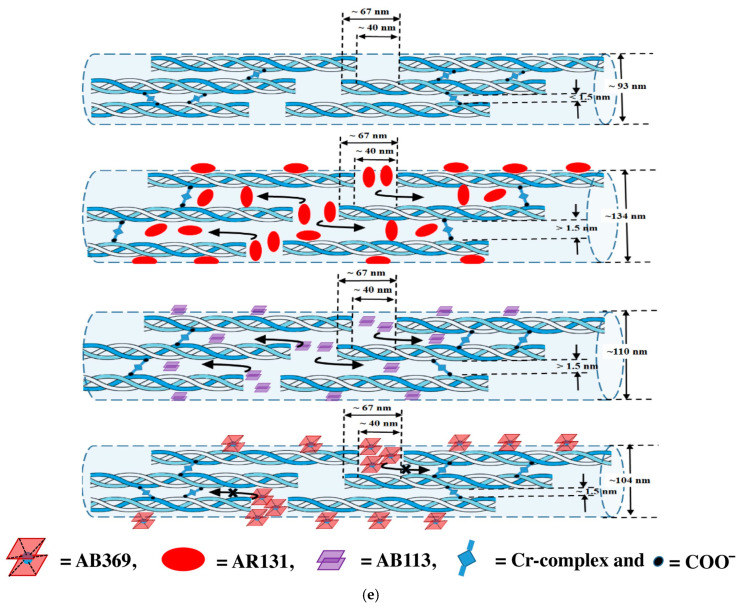
Mechanisms of dye adsorption over collagen-based materials: (**a**) Acid fuchsin and reactive blue 19 (anionic dyes) on composite COF (covalent organic frameworks)—aminated collagen fibres, abbreviated ACF-TpPa-1 [70]; (**b**) Anionic dyes (acid red and soap yellow) adsorption on multilayer ammoniated collagen fibres [38]; (**c**) Methylene blue adsorption on interpenetrating network hydrogel from (gum copal alcohols collagen)-co-poly(acrylamide) and acrylic acid [36]; (**d**) Cationic dye (methylene blue) adsorption on collagen hydrolysate (CH) crosslinked with polyvinyl alcohol (PVA) [37]; (**e**) Acid Brown 369 (AB369), Acid Red 131 (AR131) and Acid Blue 113 (AB113) removal by tanned bovine collagen fibres (TBCFs) [74].

Many studies have been carried out for dye removal from wastewater [36,37,38,39,42,61,62,63,64,65,66,67,68,69,70,71,72,73,74,75]. To increase the number of -NH_2_ groups, ethylenediamine was grafted on CF; the resulting structure was combined with TpPa-I (covalent organic frameworks) to control the porosity and stability of the adsorbent [76]. The removal mechanisms of dyes reactive blue 19 and acidic fuchsin is based on electrostatic attraction forces and π-π stacking (Figure 4a) [70]. Cross-linking collagen fibres with PEI contributed to the increasing of hydrophilic swelling of the final material; this helped to improve the adsorption capacity towards anionic dye removal (acid red and soap yellow) [38]. PEI also helped to increase the number of functional groups available on the collagen support; these groups will create electrostatic attractions with negatively charged dye molecules, enhancing adsorption (Figure 4b). The collagen surface can be modified to allow the synthesis of a hydrogel with abundant negative charges; the positively charged molecule of methylene blue (cationic dye given by the quaternary amino group) reacts with the negatively charged groups present on the adsorbent surface (Figure 4c) [36]. Similarly, Sitab et al. (2023) developed a nanomaterial using collagen hydrolysate and polyvinyl alcohol; the nanomaterial was tested for methylene blue removal, and the mechanism proceeds via the interaction of the alkyl group of the dye and of the nanofibers chain; this is followed by electrostatic interactions between the cationic dye and -COO^−^and -O^−^ groups (Figure 4d) [37]. Tanned bovine collagen fibres having macro (>50 nm), meso (2–50 nm) and micro (<2 nm) pores was used for acid dyes (acid brown 369, AB369, acid red 131, AR131 and acid blue 113, AB113) removal from wastewater; taking into account the configuration of the dye molecules, the bulkier AB369 molecule cannot pass through the micro pores, while the smaller molecules AR131 and AB113 can be adsorbed on pores of all sizes (Figure 4e) [73].

Adsorbents with magnetic properties have recently been synthesised; they have an affinity for removing target contaminants and are easily separated from water by applying a magnetic field [42,52]. The concept itself is not new; however, for collagen-based materials employed in wastewater treatment, it has only recently been tested. The percentage of contaminant removal can be controlled by changing the value of the electric field: an increase in the electric field can enhance the removal rate by up to 40% compared to when no electric field was applied [52]. When comparing the influence of electric and magnetic fields on the removal efficiency, it was found that Ni(II) adsorption is enhanced by applying an electric field compared to a magnetic field [52].

Oil sorption capacity of collagen-polydimethylsiloxane (PDMS) has been tested for silicone, vegetable and motor oil; compared with commercially available biomass materials, the collagen-based sorbent showed similar performances [77]. Modifying the collagen matrix with hexadecyl trimethyl ammonium bromide led to a slight increase in the oil sorption capacity [78].

The retention of uranyl ions on unmodified cellulose-collagen is low (Table 1) [79]. To increase the adsorption capacity, different metal ions or compounds must be added [28,80,81,82,83,84].

**Figure 5:**
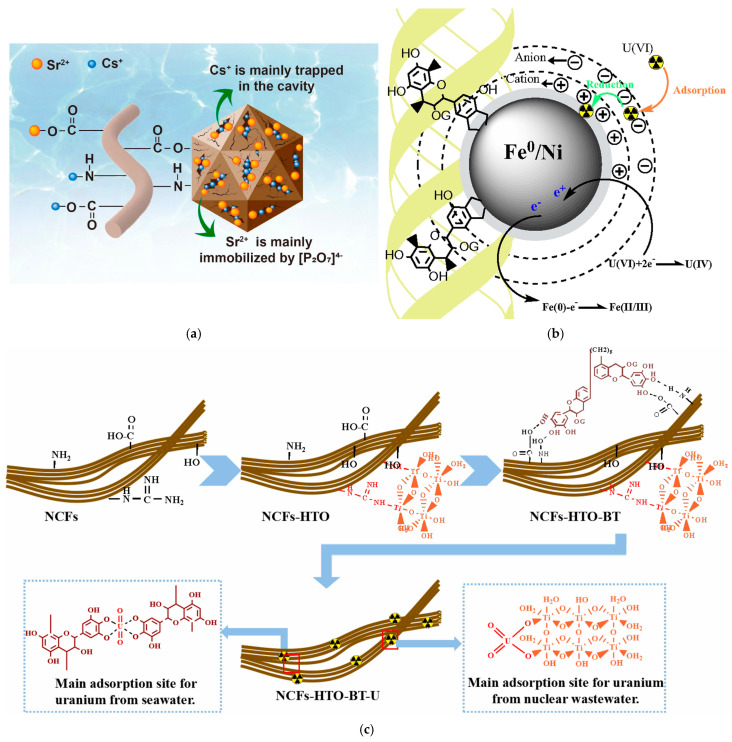
Mechanisms of radioactive ions adsorption: (**a**) Adsorption and subsequent separation of radioactive Cs^+^ and Sr^2+^ by zirconium molybdopyrophosphate-functionalized collagen fibres [85]; (**b**) Uranyl ions adsorption over nano-zero-valent Fe/Ni particles (NZFNP) loaded on collagen fibres [86]; (**c**) Uranium extraction from seawater vs. removal from wastewater NCFs—nano collagen fibrils, HTO—hydrous titanium oxide, BT—bayberry tannin [81].

The removal of Cs^+^ and Sr^2+^ from radioactive wastewater by adsorption on Zr molybdopyrophosphate-functionalised collagen fibres (ZMPPCFs) consists of ion exchange of Cs^+^ with K^+^ from ZMPPCFs while Sr^2+^ is retained by electrostatic interaction with pyrophosphate anions (Figure 5a) [85].

Liao et al. (2020) developed a material (Fe/Ni bayberry tannin/CF) used for adsorption of uranyl ions and subsequent reduction of U(VI) to U(IV) that can be easily separated further by precipitation. The mechanism consists of uranyl ions adsorption facilitated by bonds formation with bayberry tannin (BT) and collagen’s functional groups; U(VI) is reduced to U(IV) in redox reactions with Fe/Ni (Figure 5b) [86]. However, the stability of the material for reuse and its long-term performance were not discussed.

The retention mechanism of uranyl ions involves the formation of bonds (electrostatic and chelation) between the uranyl ions and the hydroxyl groups of hydrated titanium oxide, HTO (electrostatic), the ortho phenolic hydroxyl groups of BT (electrostatic) and the functional groups of collagen (-NH_2_, -COOH, -C(O)NH_2_, -OH electrostatic and chelation) (Figure 5c) [81]. *Myrica rubra* tannin also has a large number of ortho phenolic groups; immobilisation on collagen leads to obtaining a material with a high adsorption capacity of uranyl ions [82].

Titanium(IV) loaded collagen fibre has been tested for uranium removal from nuclear industrial wastewater [28,81,83]. It has been proven that HTO exhibits high affinity towards uranyl (UO_2_^2+^) extraction; however, its ability to store fluids is low [28]. This can be remediated by loading the HTO on collagen fibres that are well known for their properties to retain large amounts of water and ions and release only water in drought conditions [12]. Because of collagen ability to adsorb other ions too, the ion interference studies are extremely important. Fluoride anion usually present in radioactive wastewater gives the biggest interference (among F^−^, HCO_3_^−^, Cl^−^, NO_3_^−^, Ca^2+^, Mg^2+^ and Cu^2+^); the fluoride effect could be suppressed by the addition of Al^3+^ [28,83]. The selectivity of composite ZnS/alkali-activated collagen fibre for uranyl ions removal from a concentrated mixture of Fe^3+^, Ni^2+^, Cu^2+^, VO^3−^ and UO_2_^2+^ or K^+^, Ca^2+^, Na^+^, Mg^2+^, Cl^−^, HCO_3_^−^, SO_4_^2−^, Br^−^ and UO_2_^2+^ remains high; this is due to the combined effect of collagen’s functional groups and nano-ZnS [84].

**Figure 6:**
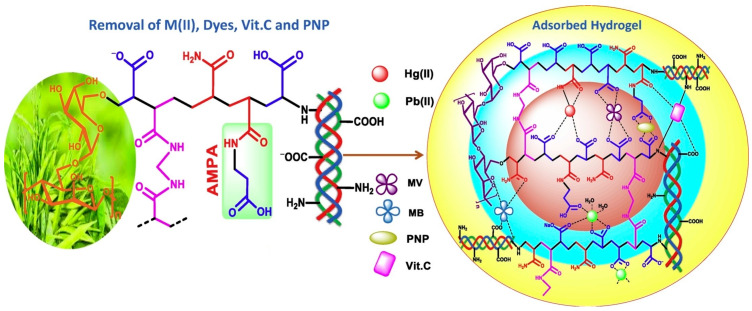
Mechanisms for simultaneous removal of heavy metals (Pb(II) and Hg(II)), dyes (methyl violet, MV; methylene blue, MB), vitamin C and p-nitrophenol (PNP) by carbohydrate and collagen-based doubly grafted interpenetrating terpolymer hydrogel (AMPA-3-acrylamido propanoic acid) [2].

Only a few studies have aimed at the simultaneous adsorption of different classes of contaminants from wastewater [2,4,5,40,87]. As a result of the variable composition of the wastewater, the adsorption materials that have been tested for the retention of several types of pollutants are preferable (e.g., wastewater resulted from dyeing also contains heavy metals) [2,87]. A collagen-guar gum-MOFs (metal–organic frameworks) composite was found to be effective for dye (methylene blue and indigo carmine—acid blue 74—with anionic character because of two sulfonate groups) and heavy metals (Ni(II), Cu(II), and Zn(II)) removal but also for neutralising the wastewater pH in some cases (wastewater resulted from cotton dyeing). Results indicated that simple collagen-guar gum hydrogel had a higher adsorption capacity for methylene blue removal than the composite material and similar behaviour in the case of indigo carmine and Ni(II). The composite material was more efficient for copper and zinc removal than the hydrogel; this is probably due to the higher uniform porosity of MOFs that allow bulkier copper and zinc internal diffusion [87]. FTIR and XPS analysis evidenced the mechanism of Hg(II) and Pb(II) adsorption on a highly functionalised adsorbent (guar gum (GG)-g-(acrylic acid (AA)-co-3-acrylamido propanoic acid (AMPA)-co-acrylamide (AM))-g-cow buffing dust (CBD)); Pb(II) preferably coordinates with -COO^−^ functional groups, mostly in bidentate chelating, while Hg(II) preferably creates bonds with the N-donor (Figure 6). Methylene blue, methyl violet, vitamin C and nitrophenol are adsorbed on the surface via hydrogen bonds, van der Waals forces and electrostatic interactions; the adsorbent showed high adsorption capacity against all tested pollutants, compared to other materials (Table 1) [2]. A collagen-based nanocomposite was also found to be extremely efficient for metal ions and dye removal [40]. Wang et al. (2022) developed an aerogel material AgNPs/Fe@CF with simultaneous activity in antibiotics’ photo degradation, antibiotic-resistant bacteria elimination and adsorption of heavy metal ions [5].

Most of the adsorption studies are oriented only towards batch adsorption experiments (static regime) that allow the understanding of the removal mechanism and determination of the adsorption kinetics and thermodynamics. There are, however, some studies that also perform continuous column experiments (dynamic column breakthrough, DCB). This is a compulsory step before large-scale utilisation of the adsorbent and gives information about the macroscopic performance of the material.

The breakthrough point for uranyl adsorption from wastewater on HTO/CF was around 250 bed volume (BV); the material in the column was regenerated using a volume of 4 BV 0.1 M HNO_3_ with a recovery rate of 98% [80]. The breakthrough point of a column packed with amidoxime/CF for uranyl removal was about 175 BV (this value was achieved in the presence of competing ions Fe^3+^, Al^3+^, Zn^2+^, Mg^2+^, Cu^2+^, Cr^3+^, VO^2+^, F^−^ and NH_4_^+^) [83]. For both materials, new cycle adsorption—regeneration gave similar results, indicating excellent reusability [80,83]. It was found that one gram of PBA (Prussian blue analog)/CF could remove more than 95% of radioactive Cs^+^ contained in four litres of wastewater (flow rate of 0.3 mL min^−1^) [88]. For highly concentrated water in uranyl ions (120.5 mg·L^−1^), ZnS/CF showed a good column adsorption performance with a breakthrough point of 75 BV [84]. The breakthrough point of a column packed with collagen fibres for thorium(IV) adsorption was 12.5 BV [89].

Compared with other materials (recycled or commercial adsorbents), collagen-based adsorbents show higher adsorption capacities for metal ions and similar adsorption capacities towards uranyl ions, dyes and oils (Table 1).

**Table 1:** Adsorption of different pollutants found in wastewater using collagen-based materials (1–47) and other materials (48–55).

	Adsorbent	Targeted Pollutant	Adsorption Capacity (at Equilibrium), mg·g^−1^	Reference
1.	CF/ZIF-7-NH_2_	Hg(II)	909.09	[57]
2.	Bayberry tannin-immobilized CF	Hg(II)	198.49	[47]
3.	Collagen-based porous fluorescent aerogel	Cr(VI)	103.3	[56]
4.	Collagen modified with oxazolidine	Cr(III)	143	[31]
5.	Carboxylated CF CF	Cr(III)	106.88 75.82	[51]
6.	Bovine hide collagen/tannin extract composite	Cu(II)	14.94	[32]
7.	Bovine hide collagen/tannin extract/sodium alginate, (SA/BHC)@TE	Cu(II)	140.56	[58]
8.	Collagen-tannin resin	Cu(II)	16.52	[54]
9.	Collagen/cellulose hydrogel	Cu(II)	67.36	[55]
10.	Manganite/collagen-polyurethane-chitosan hydrogel	Pb(II)	13.22	[34]
11.	Collagen fibre/carbon quantum dot	Pb(II)	183	[48]
12.	Persimmon tannins immobilized on collagen fibre	Ag(I)	1947	[59]
13.	AgNPs/Fe crosslinked CFs (NPs—nanoparticles)	Cr(VI) Ni(II) Pb(II)	90.734 73.82 78.19	[5]
14.	Zr—loaded collagen fibre	Cr(VI) V(V)	27.55 100.86	[50]
15.	Fish scales	Cu(II) Ni(II)	400 2.73	[52]
16.	Tannins immobilised on collagen	Cu(II) Pb(II) Cd(II) Cr(III) Zn(II)	13.30 18.41 6.5 10.4 0.8	[49]
17.	CF-PEI (collagen fibres crosslinked with polyethyleneimine)	p-arsanilic acid	285.71	[35]
18.	Hydrogel from (gum copal alcohols collagen)-co-poly(acrylamide) and acrylic acid	methylene blue	1.70	[36]
19.	Collagen hydrolysate/polyvinyl alcohol	methylene blue	99.9	[37]
20.	Black wattle tannin-immobilised mesostructured collagen	methylene blue	46.5	[75]
21.	Collagen-based cryogel, isinglass-graphene oxide	rhodamine B	120	[72]
22.	Fish scale	acid blue 113	145.3	[62]
23.	Composite hydrogels (collagen, guar gum and metal-organic frameworks)	methylene blue indigo carmine	15.8 0.46	[87]
24.	CF-PEI	soap yellow acid red (anionic dye)	538.2 369.7	[38]
25.	Tanned bovine collagen fibres	acid brown 369 acid red 131 acid blue 113	38.29 78.14 73.25	[74]
26.	Aminated collagen fibres (ACF)	acid black dye	125.63	[68]
27.	Collagen-g-poly(acrylic acid-co-N-vinylpyrrolidone)/Fe_3_O_4_@SiO_2_	methylene blue brilliant green rhodamine B	207.33 212.68 221.97	[39]
28.	Magnetic hematitenanoparticle@collagen nanobiocomposite	methylene blue rhodamine B	27.57 56.14	[42]
29.	CF	methylene blue reactive red	80 163	[61]
30.	ACF—TpPa-1	acid fuchsia reactive blue 19	257.98 449.54	[70]
31.	Zr—loaded collagen fibre Fe—loaded collagen fibre	phosphate	87.3 79.96	[90]
32.	Zirconium(IV)-Impregnated CF	fluoride	43.49	[91]
33.	Carbohydrate and collagen-based doubly grafted interpenetrating terpolymer hydrogel	Pb(II) Hg(II) methyl violet methylene blue vitamin C p-nitrophenol	976.64 859.23 116.80 58.52 212.91 59.01	[2]
34.	Collagenic-waste/natural rubber biocomposite	Hg(II) safranine brilliant cresyl blue	166.46 303.61 46.14	[4]
35.	Collagen-based hydrogel nanocomposite	Cd(II) Pb(II) methylene green crystal violet	~120 mg/g ~120 mg/g 179 652	[40]
36.	Zirconium molybdopyrophosphate-functionalised CF	radioactive Cs(I) and Sr(II)	149.52 38.99	[85]
37.	Ti(IV)/CF	UO_2_^2+^	167.4	[28]
38.	Ti(IV)/CF	UO_2_^2+^	372.4	[80]
39.	Ti(IV)/bayberry tannin/CF	UO_2_^2+^	393.19	[81]
40.	*Myrica rubra* tannin/CF	UO_2_^2+^	321.3	[82]
41.	Amidoxime/CF	UO_2_^2+^	301.18	[83]
42.	nano-ZnS/alkali-activated CF	UO_2_^2+^	359.72	[84]
43.	Cellulose—collagen	UO_2_^2+^	64.94 10^−3^	[79]
44.	Prussian blue analog (PBA)/CF	Cs(I)	175.4	[88]
45.	Collagen—tannin rearranged fibre	Th(IV)	114.97	[89]
46.	Collagen polydimethylsiloxane (PDMS)	Silicone oil Motor oil Vegetable oil	13.6 × 10^3^ 12.5 × 10^3^ 11.92 × 10^3^	[77]
47.	Collagen polydimethylsiloxane (PDMS) modified with hexadecyl trimethyl ammonium bromide	Silicone oil Motor oil Vegetable oil	15.9 × 10^3^ 14.0 × 10^3^ 12.0 × 10^3^	[78]
48.	Olive stone	Cd(II) Pb(II) Ni(II) Cu(II)	7.73 9.26 2.13 2.03	[92]
49.	Beal fruit shell	Cr(VI)	17.27	[93]
50.	Biochar	Pb(II)	46.46	[94]
51.	Biochar	Cd(II)	25	[95]
52.	Biochar	methylene blue basic fuchsin	99.11 78.01	[96]
53.	Cu_x_O/Fe_2_O_3_/MoC	reactive red 195 A reactive yellow 84	61.3 93.95	[97]
54.	nano-MgO biochar	UO_2_^2+^	333.11	[98]
55.	Recycled wool	Motor oil Vegetable oil	15.8 × 10^3^ 13.16 × 10^3^	[99]

### 2.2. Membrane and Column Separation

Wastewater purification by membranes has gained much interest due to the process efficiency in separating a wide range of compounds from wastewater (persistent organic compounds [100,101], microorganisms [102], microplastics [103,104], heavy metals [105,106]). The separation with membranes offers the advantages of metal recovery and ensures the removal of dissolved species. The extended application of membrane technologies for different classes of contaminants allows the production of good quality water that can be directly reused [107,108,109]. This combined with the ease of operation and the efforts to find new materials that are durable and cost-effective make membrane separation one of the best technologies for producing water and in some cases recovering valuable components from wastewater.

An example of industry that uses large amounts of water is petroleum processing and refining. This means that the amount of wastewater is also significant; it was reported that the processing of one barrel of crude oil generates ten times more wastewater [110] The wastewater has a complex and variable composition and includes organic matter (polycyclic aromatic hydrocarbons, benzene, toluene, xylene, ethylbenzene, phenols), heavy metals (mercury, lead, chromium Cr^6+^, cadmium, copper Cu^2+^, manganese, zinc and nickel), arsenic, As^3+^ and As^5+^, inorganic salts and oil emulsions [111,112]. However, the collagen-based membranes and column filtration materials were tested almost exclusively for emulsions separation and not for more complex wastewater mixtures. There are two types of emulsions that are discharged by the industry: oil in water (O/W) and water in oil (W/O); industrial emulsions also contain substances with the role of a surfactant.

**Figure 7:**
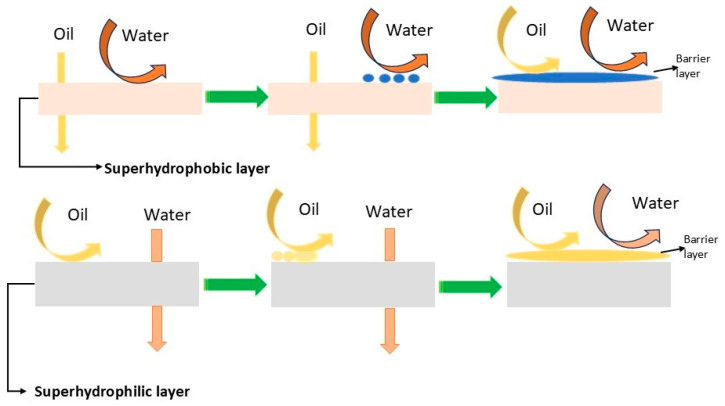
Pore clogging in conventional separation membrane (membrane fouling).

**Figure 8:**
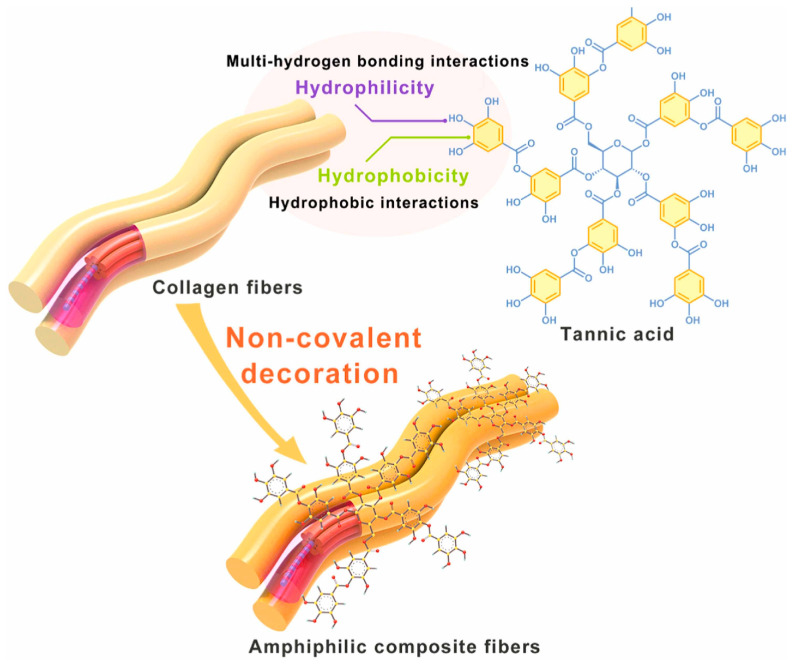
Amphiphilic character of collagen fibres [117].

Conventional separation membranes (Figure 7) have either hydrophilic or hydrophobic surfaces and are consequently subjected to rapid pore clogging (membrane fouling). Collagen has both hydrophilic and hydrophobic functional groups (amphiphilic character); this gives different wetting affinities for water and oil, allowing the separation of the emulsion [113]. The collagen fibre membrane (CFM) consists of interwoven collagen fibres that provide good mechanical resistance to the membrane [114]. CFM fails, however, in separating micro and nanoemulsions because the size of the voids between the fibres can be larger than the particle size of the emulsion droplets. To remediate this, the CFMs undergo surface modifications that improve their separation performance [115,116,117]. For instance, to enhance the amphiphilic behaviour of collagen fibres, additional compounds can be added on the surface of the collagen fibres (Figure 8). Tannic acid is a natural amphiphilic compound that can be easily deposited on the collagen fibres, improving separation [117].

Molecular dynamics simulations have shown that the driving forces allowing W/O emulsion separation are the electrostatic interactions and van der Waals forces. The anionic -COO^−^ and cationic -NH^3+^ groups of the collagen matrix generate electrostatic attraction forces towards the water molecules [113]. The oily compounds will agglomerate at the hydrophobic regions while being separated from water, which wets the surface on the hydrophilic part. The oil further permeates through the collagen fibres and is separated. This phenomenon is called underoil hydrophilicity. When the collagen fibres are wetted by O/W emulsions, the oil molecules agglomerate in the hydrophobic regions of the collagen fibres (by van der Waals forces), while the rest of the collagen fibres are wetted by the water (underwater oleophobicity) [118].

Effective separation materials consisting of blended superhydrophilic collagen fibres and superhydrophobic polypropylene fibres [119] or superhydrophilic and superhydrophobic collagen fibres [120] have been used for water recovery from O/W emulsions. Using intertwisted superhydrophobic and superhydrophilic fibres will enhance demulsification when the emulsion reaches the superhydrophilic fibres; water will be further transported along the superhydrophilic fibres while the oil will be repelled towards the hydrophobic fibres. By this method, the material fouling was delayed, and continuous separation of emulsion for 1440 min was achieved [120].

The addition of the surfactants for emulsion stabilisation also intensifies the electrostatic interactions in the separation process [107,108,109]. Industrial wastewater may contain emulsions with at least two surfactants [121]. The usual surfactants used in pharmaceutical, food, and cosmetics industries are Tween80 (non-ionic surfactant) and Span80 (biodegradable surfactant) [114,121]. Anionic or cationic surfactants can also be used (anionic: SDS—sodium dodecyl sulphate; anionic SDBS—sodium dodecyl benzene sulfonate; cationic CTAB—hexadecyl trimethylammonium bromide; cationic CPB—bromohexadecylpyridine) [107,108,109,116,117]. For an efficient reuse of water or oily compounds, the resulted aqueous filtrate should be surfactant free.

**Figure 9:**
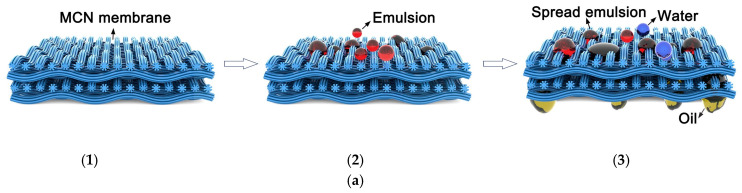

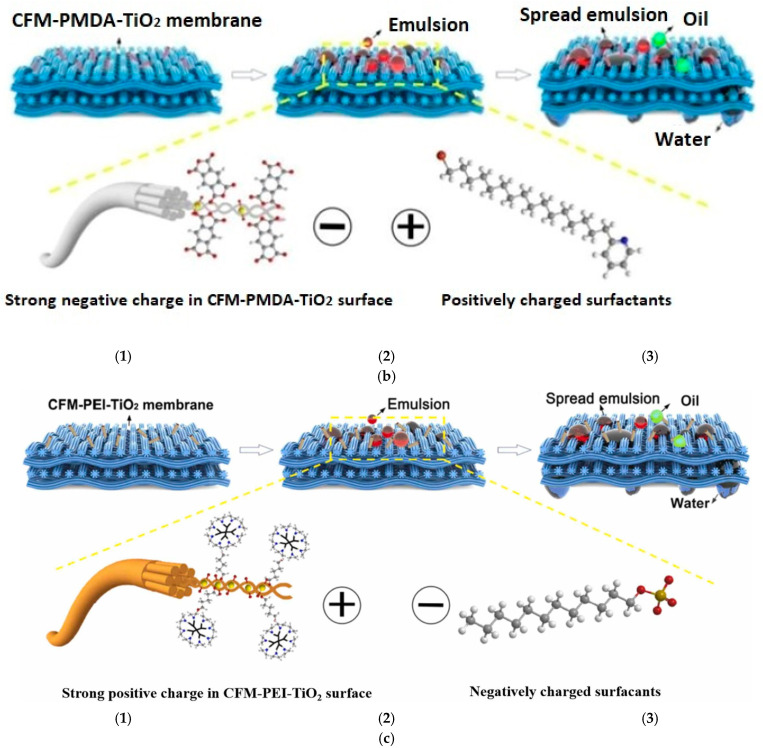
Separation of emulsion containing wastewater: (**a**) Oil—water emulsion, MCN—millimetre-scale collagen nanofibres: (1) fresh membrane; (2) emulsion spreading at the amphiprotic membrane surface; (3) separation [114]; (**b**) Cationic surfactant-stabilised oil-water emulsion, CFM-PMDA-TiO_2_ (CFM—collagen fibre mem-brane; PMDA—pyro-mellitic dianhydride) [107]; (**c**) Anionic surfactant-stabilised oil-water emulsion, CFM-PEI-TiO_2_ (CFM—collagen fibre membrane; PEI—polyethyleneimine) [108].

Ye et al. (2019) synthesised an amphiprotic membrane by deposition of amino-modified multi-walled carbon nanotubes (MWCNTs-NH_2_) and carboxyl multi-walled carbon nanotubes (MWCNTs-COOH) on a collagen nanofibres-based membrane (MCN) [114]. The separation mechanism for this consists of W/O emulsion spreading at the membrane interface (Figure 9a) followed by charge neutralisation by oppositely charged groups—-COO^−^ or -NH_3_^+^ (demulsifying). The oil is transported further through the membrane by the capillary effect along the collagen fibres.

Grafting synthetic polymers on the surface of CFM will contribute to the creation of additional charges that will enhance the emulsion separation by electrostatic interactions (e.g., polyethyleneimine, will create positive charges) [108]. The so synthesised membrane is efficient in the separation of both surfactant-free and surfactant-stabilised O/W emulsions. Adding pyromellitic dianhydride (PMDA) will increase the number of negative charges [107]. If the aim is to obtain high purity water, TiO_2_ particle deposition will amplify membrane superhydrophilicity (Figure 9b,c) [107,108]. Zr^4+^ was used to change the wettability properties of the collagen fibres matrix, obtaining a material that is superoleophobic under water (O/W emulsions) and hydrophilic under oil (W/O emulsions) [122].

The concept of size sieving membranes was used in the development of membrane that are using metal–organic framework MOFs for the emulsion separation and the collagen as a facilitator for the liquid transport along the fibres [109,115,116]. The uneven pore size distribution of CFM can be modified by incorporating MOFs with uniform pore sizes. Moreover, the pore sizes of MOFs can be customizable and can be disposed to form successive sieve-like layers to improve the separation efficiency [109]. The MOFs that have been tested consist of a zinc imidazolate framework (ZIF–8) and copper-containing MOFs (HKUST–1) [115]. Li et al. (2021) developed a double-layer collagen-based membrane CFM/UiO–66(12)/PDMS (PDMS—polydimethylsiloxane) for the separation of water in oil micro and nanoemulsions [116]. The so-called UiO 66 structure is a zirconium MOF that was set within the CFM. The principle of emulsion separation is based on the difference between the UiO-66 micropore size (which is about 6.0 Å) and the particle emulsion size, which is bigger. The superhydrophobic surface of the membrane allows the selective permeation of oil. The collagen fibres have the role of enhancing the oil transport through the membrane by the capillary effect; this has been proven by performing comparative tests on membranes with and without collagen fibres (filtrate flux of 2038 L m^−2^h^−1^ versus 866 L m^−2^h^−1^, for separation efficiencies higher than 99.99%) [114,115].

The simple collagen fibres allow a good separation of the emulsions; however, the filtrate flux is significantly lower than in the cases of modified collagen fibres or membranes. The tested membranes have proven the ability to generate high purity filtrate; they have separation efficiencies around 99% and retain the surfactant in the case of surfactant-stabilised emulsions [107,108,109,115,116,121]. Although the synthesis of collagen-based membranes is more complicated than commercial ones, the filtrate flux values are significantly higher for CFM (Table 2). The performances obtained in the case of wastewater filtering over collagen fibres are also superior to those obtained on commercial membranes (Table 2, lines 12–14).

Besides emulsions separation, CFM has been tested for acid recovery under vacuum. The recent study of Xiao et al. (2023) focused on obtaining an ecological membrane to be used for acid recovery [123]. The biocompounds collagen, casein and chitosan allowed the preparation of a thin film composite (TFC) membrane tested for the separation of acid from a solution of H^+^/Fe^2+^ synthesised to reproduce the wastewaters discharged by several industries like mining and steel production [124,125]. The mechanism that governs the protons transfer through the membrane is based on the establishment of electrostatic interactions and hydrogen bonds facilitated by the functional groups -OH, and -COOH, while Fe^2+^ ions remain at the membrane surface due to a larger ion radius and the chelation process. The performances of the TFC membrane in acid separation were found to be comparable to those obtained on commercial membranes tested in similar conditions.

**Figure 10:**
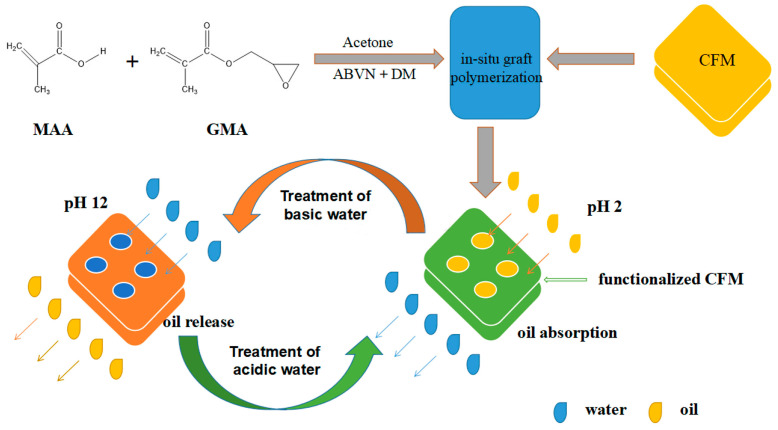
Separation of emulsion containing wastewater by pH-responsive collagen fibre material (MAA—methacrylic acid, GMA—glycidyl methacrylate, DM—dodecyl mercaptan, ABVN—2,2-azo bis(2,4–dimethylvaleronitrile)) [126].

Grafting copolymer MAA-co-GMA (MAA—methacrylic acid, GMA—glycidyl methacrylate) can create a pH-responsive separation material; the pH response is obtained by the large number of carboxyl groups provided by MAA, while GMA works as a reactive polymer creating links with MAA and collagen amine groups [126]. This can be used in the separation of emulsions (surfactant free or surfactant stabilised) as a material that absorbs or releases oil under the action of pH (Figure 10). Because of pH responsive switching wettability, the material shows exceptional anti-fouling characteristics compared with a conventional collagen-based oil absorbent [127].

### 2.3. Advanced Oxidation of Chemical Contaminants

Advanced oxidation processes (AOP) are used when the concentration of the target organic compound is low, and its recovery is not considered economically advantageous. Otherwise, alternative recovery methods such as adsorption or separation technologies are used. The main disadvantages related to AOP are the incomplete oxidation in certain cases of contaminants to compounds that are more toxic than the original ones and the accidental over-dosage of chemicals that can neutralise the effect of a subsequent biological purification step [128].

Collagen has been tested as a component of materials used in photocatalysis and Fenton oxidation. These technologies are used in the tertiary step of wastewater treatment for the removal of persistent organic compounds [129]. Photocatalysis offers the advantage of high degradation percentage [130].

Collagen is usually used as a support for catalytically active compounds due to its organised structure in fibres and fibre bundles that will lead to a catalyst with an ordered structure [131,132,133].

**Figure 11:**
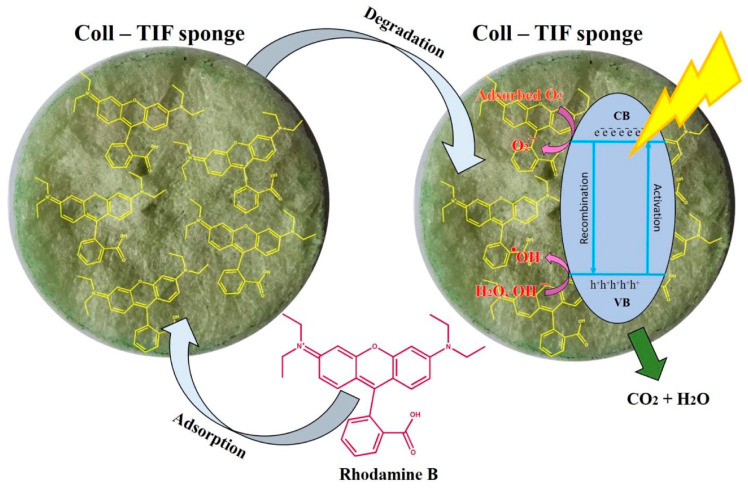
Mechanisms for pollutants removal by photo-oxidation: Rhodamine-B photocatalytic degradation over collagen-TiO_2_ nano—sponge [134].

The mechanism of crystal violet dye degradation in the presence of a collagen–cellulose–Fe_3_O_4_/TiO_2_ sponge photocatalyst consists of initial adsorption of an organic compound on the catalyst surface until the adsorption equilibrium is reached (about 12.4% of the dye is adsorbed in the first 30 min in the absence of any light source) [130]. Subsequent organic compound degradation in the presence of light contributes to the achievement of a higher removal degree (Figure 11) [130,134]. Silver chloride has been reported to be a more efficient catalyst support than TiO_2_ for the photocatalytic removal of organic compounds [135].

The high efficiency of sponge-type photocatalysts [130,134] is due to the extremely porous 3D structure that allows the easy diffusion of pollutants through the structure, determining a higher degree of adsorption of pollutants on the catalyst surface. This is adding to the material’s ability of floating at the air–water interface, which facilitates the photons transfer.

The importance of H_2_O_2_ dosage at the optimum concentration has been highlighted, an excess of hydrogen peroxide being responsible for the photocatalytic activity decrease; this is due to the formation of a hydroperoxyl radical that has a decreased oxidative activity compared with a hydroxyl radical [136]. Several silver salts (Ag_2_MoO_4_, Ag_3_PO_4_, CH_3_COOAg and AgCl) have been deposited on CFs and tested for methyl orange degradation under UV or visible light; silver chloride was found to be the most efficient under these conditions. Its efficiency was explained by the ability of AgCl to form silver nanoparticles that react with oxygen molecules to form highly reactive O_2_^−^ species [135].

Comparing the oxidation efficiency of collagen-based materials with other materials reported in the literature, it can be concluded that they represent a viable alternative for replacing more expensive materials (Table 3).

### 2.4. Antimicrobial Activity of Collagen-Based Materials

Each effluent that discharges wastewater containing microorganisms has a distinct bacterial community [141]. The groups of microorganisms present in wastewater include bacteria, protozoa, fungi and viruses [142]. While some of the microorganisms could have a beneficial effect in wastewater treatment [143], some microorganisms (e.g., Bacteroides, Clostridium, Enterococcus, Leptospira, Acinetobacter, Pseudomonas, Streptococcus, Mycobacterium [144]) are pathogens and must be monitored and removed.

Collagen’s resistance to bacterial activity makes it a good candidate for incorporation into antibacterial materials [145]. The antibacterial activity can be enhanced by additional constituents [146,147,148]. For example, adding red propolis to a chitosan/collagen membrane will give a minimum inhibitory concentration value of 7.8 μg·mL^−1^ for Staphylococcus aureus and 1.9 μg·mL^−1^ for Pseudomonas aeruginosa [146]. Ions with known antibacterial activity, like silver, can be incorporated in woven-like collagen structures, or enzymes can be immobilised on collagen surface [5,135].

**Figure 12:**
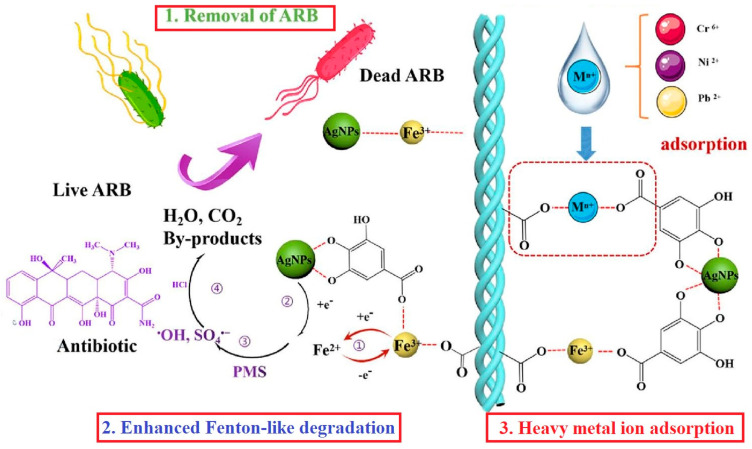
Hybrid Fenton oxidation—adsorption for simultaneous removal of antibiotics, antibiotic-resistant bacteria (ARB) and heavy metal ions (M^n+^). Peroxymonosulfate (PMS) is used as an oxidant in the Fenton process to provide hydroxyl radicals (•OH) and sulphate radicals (SO_4_^−^•) [5].

Aerogels synthesised from collagen (AgNPs/Fe@CF and Fe@CF) were tested for the simultaneous elimination of antibiotic-resistant bacteria (tetracycline resistant *E. coli*), removal of trace antibiotics by Fenton oxidation and heavy metals adsorption from wastewater (Figure 12); like in the case of sponge-type structures, the aerogel is characterised by an extremely porous structure and low density [5]. The silver nanoparticles incorporated in the collagen structure were found to be responsible for the bactericidal activity. It has been shown that silver ions adsorb on the surface of the bacterial membrane, accumulate and break the cell walls and lead to the death of the bacteria by interfering with glucose metabolism by inactivating thiol groups (-SH) [149].

Pure collagen polymers, although they do not degrade in the presence of bacteria, do not have antibacterial activity [20]. Ag_2_MoO_4_, Ag_3_PO_4_, CH_3_COOAg and AgCl, deposited on CFs, were tested for antibacterial performance; silver chloride deposited on collagen fibres was found to be the most efficient against Escherichia coli and Staphylococcus aureus, while pure collagen showed no inhibitory activity under these conditions [135].

Subbiah et al. (2022), made comparative tests of real wastewater and synthetic wastewater photocatalytic disinfection [150]. The real water was collected from the unhairing section of a tannery in Chennai (India). The synthetic water was obtained by adding *E. coli* strains (∼107 colony forming units (CFU)/mL). From Table 4, it can be seen that water containing antibiotic-resistant bacteria needs more time to achieve complete disinfection (line 1). In addition, real wastewater necessitates more time to ensure a full destruction of microorganisms (line 2); this is probably due to the presence of other chemical compounds in the wastewater that are competing for the photocatalyst active sites. However, the real wastewater was not microbiologically characterised before the experiment to know its exact composition. For comparison purposes, studies from the literature report similar or lower performances for water disinfection [151,152].

**Figure 13:**
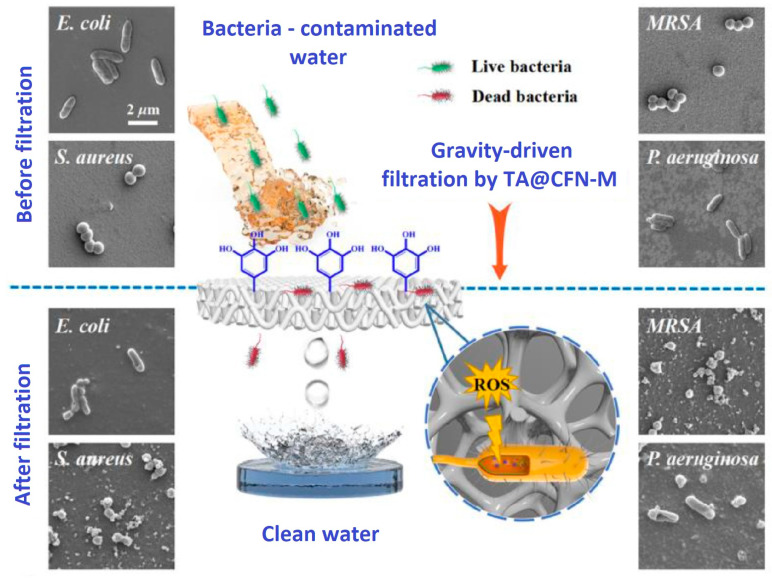
Antibacterial filtration principle, TA—tannic acids, CFN—collagen fibrous network, M—membrane, ROS—reactive oxygen species, MRSA—*methicillin-resistant Staphylococcus aureus* [153].

In addition to photooxidation, filtration of microorganisms has been used to reduce their loading in wastewater. Yu et al. (2022) tested a collagen-based membrane (TA@CFN-M, TA—tannic acids) for the microorganism filtration and wastewater disinfection [153]. The performances of this membrane were compared with those of a commercial product—mixed cellulose esters (MCE) membrane (from Millipore). While the modified collagen membrane showed good antifouling performance, the commercial membrane developed a thick biofilm layer, indicating severe biofouling due to bacteria adhering to the surface and decreased water flux. For TA@CFN-M, the *E. coli* counting in filtrate was lower than 10 CFU/mL, which is in agreement with the in force European directives for water reuse [154]. This membrane was also showing good separation performance for the retention of microplastic particles. The principle of antibacterial filtration is illustrated in Figure 13 [153]; the SEM images of four bacteria E.coli, S.aureus, MRSA (*Methicillin-resistant Staphylococcus aureus*) and *P. aeruginosa* before and after filtration were analysed. The phenolic groups of the tannic acid are providing the antibacterial effect of the membrane [155].

### 2.5. Bioremediation

Some researchers focused on the immobilisation of the enzymes on the collagen support for wastewater depollution [156,157,158]. Catalase, an antioxidant enzyme, is used in wastewater treatment; to reduce the cost of the method and increase its efficiency, it is preferred to immobilise the catalase on supports (alumina, natural polymers, synthetic polymers, etc.) [159,160]. Catalase has been immobilised on pure collagen or on modified support containing collagen [156]. Based on its ability to decompose about one million of hydrogen peroxide molecules per molecule of enzyme into water and oxygen [159], catalase supported on collagen can be used in the oxidation processes and in the aerobic bioremediation.

However, bioremediation can be applied only in specific locations where the environmental conditions allow the development of microorganisms.

The targeted contaminants for wastewater treatment using these materials are organic contaminants (e.g., dyes and bleaching effluents from the textile industry, persistent organic compounds), total nitrogen and total phosphorous [158,159,161]. Wastewater from the textile industry resulting from the bleaching process has a high amount of hydrogen peroxide. Instead of conventional use of hydrosulphite or sodium bisulphite, catalase immobilised on collagen could be used to degrade the hydrogen peroxide [159]. The resulting reactive oxygen species could be used to further degrade other organic compounds that are present in the wastewater.

Incorporation of Zr into collagen used for catalase immobilisation increased the catalase denaturation temperature from 37 to 75 °C [162], while catalase on pure collagen has a denaturation temperature of about 64 °C [156].

It has been reported that the activity of immobilised enzymes depends on the immobilisation method (cross-linking, adsorption or embedding); the activity of the enzyme immobilised by adsorption was the highest, while the addition of glutaraldehyde as a cross-linking agent improved the mechanical properties of collagen film (the elongation at break and tensile strength) and increased the denaturation temperature to about 84 °C [157].

Some natural polymers, including collagen, have been tested for bacteria immobilisation in the ANAMMOX process (anaerobic ammonium oxidation). Because of increased degradation of the natural polymer, the studies were redirected towards the use of synthetic polymers (like poly(vinyl alcohol) and poly(ethylene glycol) [163]. However, the good compatibility between the natural polymer and bacteria suggests that perhaps a compromise consisting of using both natural and synthetic polymers in the synthesis of a new support would be the best solution [164].

## 3. Regeneration and Reuse

The reusability of the material is a key factor for cost reduction and waste minimisation. In determining whether a material is feasible for large-scale use in wastewater remediation, one of the decisive factors is the ability of the material to regenerate and maintain high performance over the long term.

Most of the adsorption studies using collagen-based adsorbents were also tested to appreciate how the material behaves for pollutant desorption and for several adsorption-desorption cycles. For pollutants’ desorption, acid, bases, alcohols or salts are used.

The regeneration of CF/ZIF 7-NH_2_ by Hg(II) desorption in acid led to the decline of the adsorption capacity explained by the MOFs structural collapse determined by the ion exchange that took place between Hg and Zn ions (98.63% retention in the first cycle vs. 34.58% retention in the second one); this indicates that the material is not stable for multi-cycle use [57]. Covalent organic frameworks offer a better structural stability compared with MOFs, especially if the wastewater contains a large amount of metallic ions that are susceptible to being transferred in the MOFs structure via ion exchange [76]. Another collagen-based material tested for Hg(II) removal and exhibiting lower adsorption capacity for mercury compared to CF/ZIF 7-NH_2_ had remarkably constant removal efficiency on the tested interval (four cycles) [47]. Jing et al. (2022) developed a collagen-based fluorescent aerogel for hexavalent chromium adsorption; the regeneration of the material was performed by Cr(VI) desorption in a solution of NaOH, and the material was reused for three cycles. The removal efficiency decreased from almost 100 before reuse to about 40% at the end of the third cycle [56]. Collagen fibre/carbon quantum dot fluorescent adsorbent had an Pb(II) removal efficiency greater than 85% after five cycles [48].

Hydrogel-based collagen used for methylene blue adsorption maintained good removal efficiency for the first three adsorption—desorption cycles followed by a slow decline in the efficiency attributed to a difficult regeneration [36]. ACF-TpPa-1 maintained constant high removal efficiency for acid fuchsin during six cycles of testing, while the adsorption efficiency decreased significantly for reactive blue 19 [70].

CF/PEI maintained almost constant adsorption capacity towards anionic dyes even after five cycles of reuse; the anionic dyes were easily desorbed using an HCl solution [38]. CF/PEI effectively removes p-arsanilic acid after five times of reuse (adsorption capacity 90.20 mg·g^−1^) [35]. For five cycles of adsorption-desorption, carbohydrate and collagen-based doubly grafted interpenetrating terpolymer hydrogel had a slow decay of the adsorption capacity for Pb(II), Hg(II), methyl violet and methylene blue; the adsorption capacity for these contaminants remained, however, significant [2]. This suggests that cross-linking collagen with synthetic polymers leads to adsorbents that maintain a high adsorption capacity for a greater number of cycles. [35,38].

Similarly, zirconium molybdopyrophosphate-functionalised collagen fibres showed a high removal rate of radioactive Cs^+^ and Sr^2+^ even after four cycles [85]. Collagen/tannin extract composite had a limited decline of the adsorption capacity towards Cu(II) during six cycles of adsorption-desorption, suggesting that the material can be reused at least six times [32].

Collagen-based bio-adsorbents showed excellent reusability behaviour. Azadi et al. (2022) focused on the synthesis of bio-adsorbents from collagen and graphene oxide; this material retained a high adsorption capacity of rhodamine B even after five cycles [72]. Collagenic-waste/natural rubber biocomposite for the removal of Hg(II), safranine and brilliant cresyl blue maintained 85% from initial adsorption capacity after five cycles, suggesting good reusability [4].

Acid use for adsorbent regeneration was found to be partially responsible for the decay of the contaminants’ removal efficiency in time [40]. For a collagen-cellulose adsorbent, only the materials with a higher cellulose content were resistant in the presence of the HCl used for regeneration [55]. Another cause for adsorbent efficiency decrease is the fact that not all the adsorbate is removed during the desorption step, blocking in this way some of the adsorption centres and gradually decreasing the adsorption capacity [39].

For the adsorption of radioactive ions from nuclear wastewater (e.g., UO_2_^2+^, Cs^+^, Sr^2+^), the desorption and subsequent recovery of the adsorbate and adsorbent is even more critical from both environmental and economical points of view. However, not all the studies regarding radioactive species retention on collagen-based materials have discussed this aspect.

Most of the materials studied for radioactive wastewater treatment and reporting reusability studies demonstrated good removal of radioactive ions for at least three cycles of adsorption-desorption [28,80,83,84,85,88,165]. HTO/CF could be reused at least three times for the adsorption of uranyl from nuclear wastewater after adsorbent regeneration using nitric acid [28]. A cellulose-collagen-based biosorbent was having adsorption efficiency of 82% after five cycles of adsorption-desorption of uranyl ions, suggesting a high degree of reuse [79].

The materials used for oil sorption were regenerated by centrifugation; it was found that their sorption capacity towards silicone, motor or vegetable oil is slowly decreasing during five cycles but is kept above 93% of the initial sorption capacity; however, not all the oil could be removed from the sorbent by centrifugation, and another desorption method must be provided [78].

Emulsion viscosity was found to have an influence on the number of reuse cycles on membranes, filtration materials or adsorbents because of severe pore blockage by the viscous liquid [114,121,126]. CF material modified by copolymer P(MAA-co-GMA), has a decrease in its efficiency after the sixth reuse cycle in stabilised motor oil/water emulsion separation [126]. For membranes, this disadvantage can be mitigated by passing to vacuum filtration. The separation fluxes of nano-emulsions water-in-olive oil and water-in-pump oil under gravity effect were less than 10 L·m^−2^·h^−1^, while under vacuum, the filtrate fluxes were above 1299 L m^−2^·h^−1^ bar^−1^ [121].

Most membranes and filtration materials tested for emulsion separation maintain their separation efficiency with no significant decline in the filtrate flux [107,108,109,115,117,120]. Ye et al. (2024) tested the filtrate flux stability of CFM-PEI-TiO_2_ membrane over 120 min of continuous emulsion separation. It was found that the separation efficiency decreased from 99.9 to 99.2%, while the filtrate flux increased from 1351.92 L·m^−2^·h^−1^ (Table 2) to more than 1550 L·m^−2^·h^−1^ [108].

Collagen/casein/chitosan polymer membrane used for acid separation from wastewater showed excellent reusability for five cycles of testing [123].

Collagen fibres material containing Zr^4+^ was found to have a 5% decrease in the filtrate flux after 120 min of continuous use, which was attributed to the contamination of the separation material with oil and surfactant [122].

The regeneration of filtration material/membrane can be achieved by washing with ethanol and subsequent drying at about 40 °C; the material/membrane is then ready for the next continuous separation cycle (120 to 150 min) [107,120]. In this way, the oil is removed from the surface of the separation material. It has been reported that a membrane maintained its high-water filtrate flux even in the sixth cycle of reutilisation (separation efficiency up to 99.98, water filtrate flux 1804 L·m^−2^·h^−1^) [117].

Liu et al. (2009, 2010), discussed the reutilisation of a Fe(III) immobilized on CFs photocatalyst, used for malachite green and orange II removal [133,136]. A slight decrease in the photocatalytic activity after each cycle of reutilisation has been reported. This was attributed to the decrease in the adsorption capacity of the catalyst and to the loss of iron. To reactivate the catalyst, the Fe(III) reimmobilisation onto the catalyst has been considered.

The efficiency of tetracycline removal by photocatalytic degradation in the presence of AgNPs/Fe@CFs after five cycles was higher than 90%; the same material was tested for the elimination of tetracycline resistant *E. coli*, and its elimination rate after five cycles was very high (99.99%) [5].

When retaining oil on collagen-cellulose-Fe_3_O_4_/TiO_2_ (sponge-type structure), it was proven that this material can be used up to at least nine cycles without a significant decrease in the degree of oil removal from water [130].

Catalase immobilised on collagen was tested for hydrogen peroxide decomposition, and it was found that the material can be reused up to 45 times when working in optimal conditions (temperature 30 °C, pH = 7) [156].

## 4. Environmental Implications

Although collagen toxicity is small and high quantities of collagen must be ingested before some health effects can be observed [19], the final material that is used in wastewater treatment may contain substances that are not harmless (e.g., silver, iron, synthetic polymers, etc.). Therefore, advanced studies of stability over time and degradation under the action of water pH and temperature are required before using these materials on a large scale. Collagen-based materials can be susceptible to proteolytic degradation (protein breakdown into smaller polypeptides or the corresponding amino acids); the addition of, e.g., magnetite improves the resistance of the material to acidic and proteolytic degradation [34].

In addition, the material must have good mechanical stability, so that it is not entrained, blocking the equipment and the pipes through which the water passes in the subsequent stages of decontamination. For membrane materials, some authors report performing abrasion stability tests [107,108,109,116,121]. These tests consist of membrane abrasion with sandpaper followed by contact angle checking at various intervals. The membrane is then reused for emulsion separation experiments, and its performance is reevaluated. For example, it was found that CFM-PEI-TiO_2_ surface superhydrophilicity has been kept even after 500 cycles of abrasion [104]. CFM/UiO-66(12)/PDMS membrane maintained its high performance in the separation of six types of micro and nano-emulsions, after 200 abrasion cycles (oil separation flux up to 1012.2 L m^−2^ h^−1^, efficiency up to 99.993%) [116].

Generally, the materials that are used in wastewater treatment must have a good resistance to degradation in a harsh chemical environment given by the presence of organic solvents, strong acids or strong alkali. The chemical stability can be tested by membrane immersion for 2 h in strong acid (pH = 2) and alkali (pH = 12) solutions [109,116]. The resistance towards the corrosive environment was attributed in the case of UiO-66-NH_2_ membrane to the supplementary membrane functionalisation with -NH_2_ groups [109]. The stability of a thin film composite (collagen, casein, chitosan) membrane used for acid recovery was evaluated by performing acid immersion tests for extended time (10 days); although some mesopores have been formed in the membrane, it has been reported that the membrane overall performance was not impacted significantly [123]. Li et al. (2021) also evaluated the resistance towards organic solvents (e.g., by immersion in dodecane, n-heptane or n-octane solution); the tested materials were reassembled, and emulsion separation experiments indicated that the materials kept their separating performances [116].

The stability of AgNPs/Fe@CFs aerogel used for antibiotic degradation, antibiotic-resistant bacteria elimination and heavy metals adsorption was evaluated by estimating silver leaching corresponding to each cycle; after the fifth cycle, the cumulative silver leaching was about 0.13%, which corresponds to a concentration of silver in water significantly lower than the maximum concentration allowed in the drinking water standards [5].

Collagen crosslinked with synthetic polymers was found to have better performance in acidic conditions or at high temperatures [33,38].

When foreseeing adsorption utilisation for wastewater treatment, the stability of the material to irradiation must be evaluated. This is done by performing morphological analysis and identification of the functional groups (SEM and FTIR) of the adsorbent before and after irradiation at different doses. In this regard, Tang et al. (2021) found that there is a slight decrease in the adsorption capacity of HTO/CF after the first ^60^Co γ-ray irradiation dose, followed by constant adsorption capacity when the irradiation dose increases [80]. PBA/CF showed also good irradiation stability after exposure at different doses of ^60^Co γ-ray irradiation (10–350 kGy) [88]. Fe/Ni loaded on bayberry tannin/CF showed adsorption capacity decrease with increasing irradiation dose, but the removal rate remains high (above 98%) [86].

Some adsorbents are intended to be biodegradable [36,45,56]. The adsorbent material synthesised by Kaur and Jindal (2020) is ecofriendly; this adsorbent biodegrades under the action of microorganisms by metabolic and enzymatic processes specific to soil burial or composting methods [36,45]. Du et al. (2018) compared the biodegradability of CF-PDMS with that of simple CF during a soil burial test performed for 75 days and found that the PDMS modified material has a weight loss above 52% after 75 days, while CF loses about 67% of its initial weight [78].

Ye at al. (2023) report biosafety tests for CFM-PMDA-TiO_2_ [107]. The toxicity of the membrane was evaluated by studying zebrafish evolution in the filtrate or in the water that contains the membrane; the zebrafish population remained unaffected after 14 days of testing, both tests indicating that the membrane is not toxic.

## 5. Challenges and Conclusions

Experiments with real wastewater are tremendously useful to appreciate the behaviour of the materials when competitive adsorption takes place and if the affinity of the materials towards the tested pollutants shifts to water components that are not necessary to be removed (e.g., Ca^2+^, Mg^2+^, K^+^, Na^+^, Cl^−^, etc.). There are a limited number of studies with real wastewater [35,58,87,107,150]. To overcome this aspect, some researchers have performed ion interference studies [32,56,58,80,81,84].

The Hg(II) retention on CF/ZIF 7-NH_2_ from water contaminated with multi ions (potassium, magnesium, calcium, cadmium, manganese) evidenced the high affinity of the adsorbent towards Hg ions (315.89 vs. 332.1 mg·g^−1^ in the absence of competing ions) [57]. In the presence of other ions (trivalent iron, bivalent copper, barium and lead), it was found that the Cr(VI) removal performance of a collagen-based aerogel decreases from almost 100% to about 65%, hence, emphasising the importance to study the performances of the materials in conditions that are similar to real ones [56]. In general, for retention mechanisms that are based on coordination between metal ions and organic functional groups (little electrostatic influence), the competition for retention will be given by metal ions that have a similar hydrated radius [88]; when electrostatic interaction is predominant, the interference in the retention will be given by the ions that have the same valence as the target ion [32,58].

The removal efficiency of As(III) using Zr/CF was highly influenced by coexisting anions like Cl^−^, NO_3_ ^−^, SO_4_^2 −^ and HPO_4_^2−^, dividing the removal efficiency by 2. On the contrary, low concentrations of HCO_3_^−^ do not affect the As(III) adsorption [60].

p-arsanilic acid was added to river or lake water samples to study the removal of this contaminant on amphiphilic amino-modified CF; the contaminant concentration in the treated water was less than the maximum allowable concentration [35].

**Figure 14:**
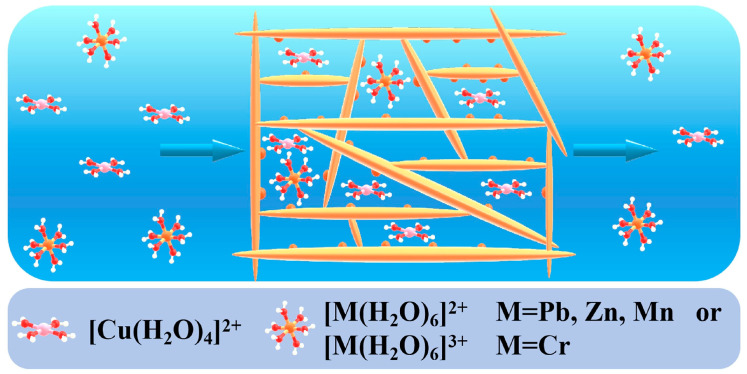
Influence of other species present in the wastewater [58].

Antibiotics and other metal ions have also been found to compete for adsorption sites when removing heavy metals from water; the efficiency of Cu(II) removal of (SA/BHC)@TE decreased from 90.3% to 80.07% in the presence of tetracycline (Figure 14) [58]. To better understand, the authors did comparative experiments with tap water, sea water and lake water; the lowest Cu(II) removal efficiency (76.32%) was recorded for sea water due to increased water mineralisation [58].

Some researchers preferred to perform the adsorption kinetics studies using methylene blue and indigo carmine, while the reusability of the adsorbent was tested for 10 cycles on real wastewater resulting from the dyeing stage (cotton dyeing—containing direct red and mordant brown; acrylic fibre dyeing—basic pink dye); the adsorbent was regenerated after each adsorption cycle using NaCl and had a removal efficiency greater than 70% for the first eight cycles [87].

Collagen-graphene oxide nanocomposite was tested for dye and phenolic compounds removal from tannery effluents; the material proved to be efficient, reducing the COD (chemical oxygen demand) and BOD (biochemical oxygen demand) values below the limits imposed by the wastewater directive [73].

The efficiency of nano-ZnS/CF for uranyl removal out of lake and river water samples was found to remain high (86.64% and 92.89%) [84]. The experiences with synthetic nuclear wastewater showed a good selectivity of adsorbents towards uranyl ions; the common compound of the tested materials was collagen, and the selectivity for uranyl ions was attributed to the strong bonds that uranyl ions create with the collagen’s functional groups [28,80,81,83,84,165].

Separation on membranes can be complicated by the presence of a mixture of several oily compounds instead of an emulsion formed of one oily compound in water. Ye at al. (2023, 2024) performed separation studies using emulsions prepared from commercially available cosmetics (facial creams, shampoos, hand creams, hair conditioners) to simulate real wastewater resulting from the cosmetics industry [107,108]. The results can be compared with the separation efficiencies and filtrate fluxes obtained from tests with a single oil/water emulsion on the same membrane. For CFM-PMDA-TiO_2_ membrane, separation efficiency remains higher than 94%; the filtrate flux decreases due to the complex mixture of oily compounds present in the cosmetic wastewater. The filtrate flux remains high anyway (579.9 L·m^−2^·h^−1^ in the worst case [107]). The CFM-PEI-TiO_2_ membrane gives, however, remarkably better performances then the CFM-PMDA-TiO_2_ membrane (the efficiency when separating cosmetic emulsions was above 98.5%, minimum filtrate flux 985.0 L·m^−2^·h^−1^, maximum filtrate flux 1486.4 L·m^−2^·h^−1^) [108]. This highlights the importance of additional compounds that are included in the collagen membrane (PEI vs. PMDA).

An aspect that was neglected is the calculation of the cost of the material. Although collagen is recovered from waste, this generates a cost, which is added to the production cost of the final material. Peng et al. (2023) reported a small cost compared with other materials (0.027 $/gram for zirconium molybdopyrophosphate-functionalised collagen fibres vs. other industrial adsorbents for nuclear wastewater that are in the range of 0.133–18.821 $/gram) [85]. In addition, Yang et al. (2024), reported smaller manufacturing costs for their adsorbent compared with the price of industrial adsorbents for nuclear industry [84].

The purpose of this review was to explore the state of research on the use of recovered collagen as a sustainable alternative for wastewater treatment. It is rarely that wastewater is loaded with only one type of pollutant. In general, it is a mixture of different types of contaminants such as heavy metals, organic substances (e.g., cosmetics, pharmaceuticals, surfactants, oils, dyes, etc.) and acids. The collagen-based materials have proven their effectiveness in retaining a wide range of organic and inorganic compounds from the classes above mentioned, as well as in retaining isotypes from the wastewater resulting from nuclear power plants.

The current methods that have been tested for wastewater purification using collagen-based materials are adsorption, photooxidation, separation using membranes or bed filtration and coagulation-flocculation. Without a trace of doubt, collagen has proven its effectiveness in the depollution of wastewater; it can be incorporated into various materials used in many of the technologies specific to wastewater treatment plants.

Most of the adsorption studies also address the issues of material stability and reusability. The positive results of the tests performed to evaluate the irradiation stability of collagen-based materials together with the experiments in a dynamic regime suggest that collagen adsorbents are good candidates for the treatment of radioactive wastewater. The ability of the membranes to withstand mechanical stress was evaluated, and it was found that the collagen membranes generally maintain their wetting properties even after a large number of abrasion cycles. Since these materials are intended for use in wastewater treatment plants, it means that they are exposed to a harsh chemical environment. Therefore, their reusability and stability must be properly evaluated before implementing the use of these materials on a larger scale.

Despite its proven antibacterial activity, few studies have been dedicated so far to collagen-based materials used for microorganisms’ removal from wastewater. These aspects must be studied more in depth considering the excellent antimicrobial properties of collagen-modified materials and the possibility of coupling good adsorption capacity with microorganism reduction.

The removal of microplastic by filtration on collagen-based materials requires advanced deepening. So far, only one study addresses the issue of microplastic retention by filtration on collagen-containing membranes.

Another aspect that needs to be highlighted is that with a few exceptions, most of the studies have been performed on synthetic wastewater samples. Some authors have studied, however, the interference of other contaminants or species in the removal process of the targeted compound.

## Figures and Tables

**Table 2:** Performance of different collagen-based fibres (1–5) and membranes (6–14) for wastewater treatment.

	Filtration Material/ Membrane	Targeted Pollutant	Separation Efficiency %	Filtrate Flux L·m^−2^·h^−1^ *	Reference
1.	Amphiphilic composite fibres—tannic acid onto collagen fibres (ACFs)	Heptane/Water Kerosene/Water Dodecane/Water Octane/Water (SDBS) Water/Heptane (Span80) Water/Kerosene (Span80) Water/Dodecane (Span80) Water/Octane (Span80)	>99.99	1911 (W) 1745 (W) 1822 (W) 1720 (W) 2166 (O) 2471 (O) 1936 (O) 2408 (O)	[117]
2.	Collagen(I) Fibres (CF)	n-Dodecane/Water Kerosene/Water Petroleum ether/Water Water/n-Dodecane (Span80) Water/Kerosene (Span80) Water/Petroleum ether (Span80)	>99.99 99.987 99.989 >99.99	257.3 (W) 313.4 (W) 262.4 (W) 2929.9 (O) 2738.5 (O) 3337.6 (O)	[113]
3.	Zr^4+^ supported on Collagen(I) fibres	Dodecane/Water Olive oil/Water Pump oil/Water Water/Dodecane Water/Kerosene Water/Petroleum ether	>99.99	3031 (W) 2407.6 (W) 2598.7 (W) 2331.2 (O) 2751.6 (O) 2216.6 (O)	[122]
4.	CFs-PPFs (PPF—polypropylene fibres, dual-channels) Simple collagen fibres	Dodecane/Water Dodecane/Water	>99 99.4	2844 (W) 434 (W)	[119]
5.	SCFs-DC (superwetting collagen fibres with water-oil dual-channels) Simple collagen fibres	Dodecane/Water Kerosene/Water Hexadecane/Water Octane/Water Dodecane/Water	98.99 99.10 99.04 96.22 99.45	666 (W) 587 (W) 487 (W) 796 (W) 477 (W)	[120]
6.	CFM-PMDA-TiO_2_ (CFM—collagen fibre membrane; PMDA—pyromellitic dianhydride)	Dodecane/Water (CTAB) Heptane/Water (CTAB) Octane/Water Dodecane/Water (CPB)	98.35 98.70 99.86 97.28	600 (W) 900 (W) 1436.40 (W) 1100 (W)	[107]
7.	CFM-PEI-TiO_2_	Dodecane/Water Hexane/Water (SDS) Dodecane/Water (SDBS) Hexane/Water (SDBS) Heptane/Water (SDS) Heptane/Water (SDBS)	99.93 98.79 99.83 99.73 99.94 99.68	988.90 (W) 880.75 (W) 1351.92 (W) 1346.09 (W) 1148.58 (W) 1458.50 (W)	[108]
8.	UiO-66-NH_2_ membrane incorporating MOFs	Nanoemulsions Dodecane/Water (SDS) n-Hexane/Water (SDS) n-Octane/Water (SDS) n-Hexadecane/Water (SDS) Microemulsions Dodecane/Water (SDBS) n-Hexane/Water (SDBS) n-Octane/Water (SDBS) n-Hexadecane/Water (SDBS)	>99.8 >99.7	217.25 (W) 242.12 (W) 210.86 (W) 308.46 (W) 200.66 (W) 225.01 (W) 370.05 (W) 291.87 (W)	[109]
9.	CFM/UiO-66/PDMS (PDMS—polydimethylsiloxane)	Nanoemulsions Water/Dodecane (SDBS) Water/Dodecane (CTAB) Water/n-octane (SDBS) Water/n-heptane (SDS) Microemulsions Water/Dodecane (SDS) Water/Dodecane (CTAB)	>99.99	540.4 (O) 504.6 (O) 969.8 (O) 973.3 (O) 545.4 (O) 351.1 (O)	[116]
10.	CF/ZIF-8/PDMS, (ZIF—zinc-based MOFs)	Nanoemulsions Water/Dodecane (SDBS) Water/Dodecane (CTAB)	>99.99	1982 (O) 1809 (O)	[115]
11.	MWCNTs/CFM (MWCNTs—multiple-walled carbon nanotubes)	Water/Heptane	>99.99	1051 (O)	[121]
12.	Commercial PTFE (polytetrafluoro ethylene)	Nanoemulsions Water/Dodecane (SDBS) Water/n-heptane (SDS)	>99.99	77.6 (O) 305.6 (O)	[116]
13.	Commercial double-sided polyvinylidene fluoride (PVDF) Commercial PTFE	Water/Dodecane (SDBS) Water/Dodecane (SDBS)	>99.99	51 (O) 95 (O)	[115]
14.	Commercial polyamide Commercial PTFE	Water/Heptane	>99.99	64 (O) 35 (O)	[121]

* (O)—oil; (W)—water.

**Table 3:** Performance of collagen-based (1–7) and other materials (8–11) for pollutants removal by photocatalytic oxidation.

	Photocatalyst	Targeted Pollutant	Removal Degree (%)	Degradation Time (min)	Light Source	Reference
1.	Collagen-cellulose-Fe_3_O_4_/TiO_2_	Crystal violet	91.2 86.6	180 180	H_2_O_2_, Visible light (200 W Hg (Xe)) H_2_O_2_, direct sunlight irradiation	[130]
2.	CFs-TiO_2_	Rhodamine B	95 73 100	130 130 300	H_2_O_2_,Visible light (200 W Hg (Xe)) H_2_O_2_, direct sunlight irradiation	[134]
3.	Fe(III)/CFs (CFs-collagen fibres)	Orange II p-nitrophenol	~100 95	20 20	H_2_O_2_, UVC irradiation (254 nm, 8 W or 4 W)	[132]
4.	Fe(III)/CFs	Orange II	73.8	90	H_2_O_2_, UVC irradiation (254 nm, 10 W)	[136]
5.	Fe(III)/CFs	Malachite green	55	120	H_2_O_2_, UVA irradiation (365 nm, 10 W)	[133]
6.	AgCl/CFs	Methyl orange	80 >90	210 30	UV light (370 nm, 36 W) visible light (500 W Xe lamp)	[135]
7.	AgNPs/Fe crosslinked CFs (NPs—nanoparticles)	Mixture of antibiotics (Tetracycline hydrochloride, sulfamethoxazole, ciprofloxacin, vancomycin and levofloxacin)	>90	30	H_2_O_2,_ direct sunlight	[5]
8.	Cu-doped nanosized ZnO	Methylorange	85	200	Direct sunlight	[137]
9.	Zn-doped CdS	Rhodamine B	93	135	Visible light	[138]
10.	CoO/TiO_2_	Rhodamine B	97	120	Infrared light irradiation	[139]
11	BiOI (bismuth oxyhalide)	Reactive blue 19	95	120	Direct sunlight	[140]

**Table 4:** Performance of collagen-based (1–2) and other materials (3–4) for wastewater disinfection under the action of light.

	Material	Microorganisms	Removal Degree (%)	Inactivation Time (min)	Light Source	Reference
1.	AgNPs/Fe crosslinked CFs (NPs—nanoparticles)	Tetracycline resistant *E. coli* Methicillin-resistant *Staphylococcus aureus*	99.99 99.99	60 60	H_2_O_2,_ direct sunlight	[5]
2.	Ag-SiO_2_/amino-functionalised collagen	Tannery wastewater disinfection *E. coli* containing wastewater	100 100	20 15	Visible light (200 W Hg (Xe))	[150]
3.	Ag/CuS/carbon cloth	*Bacillus subtilis*	99.99	22.5	Visible light (500 W, Xe)	[151]
4.	Ag/polymeric carbon nitride	*E. coli*	95.5	120	Visible light	[152]
